# SafetyNet: streamlining and automating QA in radiotherapy

**DOI:** 10.1120/jacmp.v17i1.5920

**Published:** 2016-01-08

**Authors:** Scott W. Hadley, Marc L. Kessler, Dale W. Litzenberg, Choonik Lee, Jim Irrer, Xiaoping Chen, Eduardo Acosta, Grant Weyburne, Wayne Keranen, Kwok Lam, Elizabeth Covington, Kelly C. Younge, Martha M. Matuszak, Jean M. Moran

**Affiliations:** ^1^ University of Michigan Department of Radiation Oncology Ann Arbor MI; ^2^ Varian Medical Systems Palo Alto CA USA

**Keywords:** quality assurance, patient safety, automation

## Abstract

Proper quality assurance (QA) of the radiotherapy process can be time‐consuming and expensive. Many QA efforts, such as data export and import, are inefficient when done by humans. Additionally, humans can be unreliable, lose attention, and fail to complete critical steps that are required for smooth operations. In our group we have sought to break down the QA tasks into separate steps and to automate those steps that are better done by software running autonomously or at the instigation of a human. A team of medical physicists and software engineers worked together to identify opportunities to streamline and automate QA. Development efforts follow a formal cycle of writing software requirements, developing software, testing and commissioning. The clinical release process is separated into clinical evaluation testing, training, and finally clinical release. We have improved six processes related to QA and safety. Steps that were previously performed by humans have been automated or streamlined to increase first‐time quality, reduce time spent by humans doing low‐level tasks, and expedite QA tests. Much of the gains were had by automating data transfer, implementing computer‐based checking and automation of systems with an event‐driven framework. These coordinated efforts by software engineers and clinical physicists have resulted in speed improvements in expediting patient‐sensitive QA tests.

PACS number(s): 87.55.Ne, 87.55.Qr, 87.55.tg, 87.55.tm

## I. INTRODUCTION

Quality assurance (QA) is an essential task in radiation oncology that keeps our patients safe^(1)^ but results in rework when a plan fails QA tests and can lead to inefficiencies in patient care. QA is also time intensive and often requires physicists, dosimetrists or other staff to manually import and export data, review parameters, collect measurements, and approve results. QA tasks often wait until someone is available to execute these manual steps. A major drawback of manual tasks is that they can be executed incorrectly, which corrupts the process and leads to delays and possible errors. Time pressures in radiotherapy can lead to reduced vigilance and cause QA steps to be delayed or skipped, potentially resulting in catastrophic outcomes.^(2)^ Radiotherapy made a big leap in patient safety with the implementation of record and verify and computer‐controlled treatment delivery.^3^, ^4^ Automatically sending the correct plan information to the treatment machine for delivery and returning the treatment actuals back to the R&V system reduced or eliminated the chance for human errors by minimizing the manual steps required to treat a patient.^5^, ^6^, ^7^

In this work we present our efforts to bring the same level of rigor that exists in treatment delivery to the other areas of radiotherapy. We automate QA by eliminating the manual steps from the process with automated software agents to reduce human errors. This work was influenced by our Lean Thinking^8^, ^9^ efforts that seek to reduce waste. An event‐driven framework is added to the oncology information system (OIS) to trigger QA off of key milestones in the radiotherapy process.^(8)^

## II. MATERIALS AND METHODS

Ideas for new software projects come from the clinical needs identified by the medical physicist working with the treatment team. The physicist determines the scope of the project and requirements for a successful QA tool. A project starts after a high‐level review committee determines that the cost and benefits of the project are worth expending software engineering effort. At that point a small team of one or two physicists and engineers is formed to complete the scope and requirements of the software. The teams are based on the clinical need and the specific experience and background of the engineer. Software requirements determine the data inputs and outputs and user interface if needed. A hazard and risk analysis is done for those software tools that could impact patient treatment if they did not perform as specified. Those hazards are mitigated either by changing the function of the software or having a review of the results by the user.

**Figure 1:**
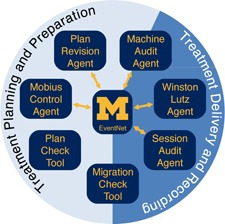
Overview of SafetyNet system. EventNet is central to the operation of the software agents, which receive events to activate QA and send messages notifying of results.

Figure 1 is a diagram of the SafetyNet system. The following sections contain short descriptions of key software tools that have been developed to achieve the department's goals of safe and efficient plan production, QA, and treatment delivery.

### A. EventNet

EventNet is an in‐house‐developed message brokering platform for radiotherapy that utilizes a publish‐subscribe (pub‐sub) architecture. In a pub‐sub architecture, publishers send events to the message broker, and the broker forwards those events to all subscribers who have signed up for notification. For this work, events are user‐defined and represent significant changes to data or the process used for radiotherapy planning and treatment delivery. Table 1 lists some of the most common events brokered by EventNet. The brokered pub‐sub architecture decouples publishers and subscribers so they can evolve independently. Publishers and subscribers are coupled only to message definitions and the broker's publish/subscribe interfaces.

**Table 1:** Partial List of events brokered by EventNet. The event finder generates messages when these and other attributes change status in the OIS.

*Event Name*	*Description*	*Status Change*
Plan Status	Plan status in Eclipse/Aria has changed from one status to another.	Unapproved, Reviewed, Planning Approved, Treatment Approved, Unplanned
Treatment Session Status	Treatment session has changed indicating a treatment in progress, or completed treatment.	Open, In Progress, Partial Complete, Complete, First Session Complete, Last Session Complete.
Appointment Check‐In	A patient has been checked in for their appointment.	Checked in for Appointment
Scheduled Activity	A treatment or other appointment has been created or date changed in the OIS.	Created, Moved or Canceled

**Figure 2:**
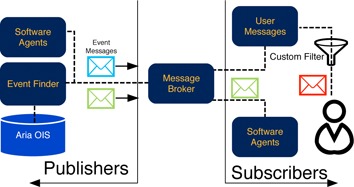
Diagram of EventNet interaction and function. The event finder watches the ARIA OIS and generates events for EventNet to process. Likewise software agents can subscribe and publish their own events via EventNet.

EventNet consists of three parts (diagramed in Fig. 2): event publishers, the message broker, and event subscribers. The main event publisher in the system, Event Finder, monitors the ARIA OIS (Varian Medical Systems, Palo Alto, CA) by way of an SQL query for domain‐relevant changes of interest. This query is refreshed every 30 s and compared to the previous query results for state changes in the data. When changes are detected, Event Finder publishes the change to the message broker as an event. Some of the events we have implemented include: Patient Check‐in, Plan Status Change, Treatment Session Status Change, and approximately 20 others.

Software agents are tools that run independently of user interaction and can be both event subscribers and event publishers. A typical software agent is one that performs a QA check that results in a “QA passed” or “QA failed” event based on its programmed rules. Medical physicists could subscribe to the “QA failed” event and receive immediate notification by means of an email, text message or page. A Web‐based interface was developed to allow users to easily subscribe to events, to set the medium for notification, and to apply basic filtering to select the events for notification. All of this is running on a single server that is separate from the OIS.

EventNet has been running against our clinical database since August 2012. It has undergone continuous refinement to improve its function and to satisfy evolving use cases elucidated by the clinic. It was released for routine clinical use in October of 2014 and has become an integral part of our clinical work. Approximately 3,000 unique events are processed by the system each day in our department, which operates six linacs that treat, on average, 131 different plans daily. Within dosimetry, an average of 10.5 new plans are created and 0.65 plan revisions occur each day.

### B. Plan Revision Agent (PRA)

The Plan Revision Agent was designed to improve the safety and efficiency of reviewing copied and revised treatment plans. Plan revisions are created when a change is applied to a plan already under treatment. Many plan changes and revisions are requested urgently, making the safety and efficiency of a check critical. Since the plan revision creates a copy of the plan, the proper copying of the existing data and changes must be verified. Parameters checked include dose and dose per fraction information, plan settings including each leaf position regardless of the number of control points, and accessories. An example revision includes a change to add more time for a treatment field using the field‐in‐field technique or a minor adjustment of the jaw settings for a tangent for a breast patient once confirmed that the monitor units are not changed. For intensity‐modulated treatments, such a plan revision would require measurement‐based QA prior to treatment. We estimate that for these plans, we save approximately 1.5 to 2.5 hr of time because pretreatment measurements do not need to be repeated and reviewed, and instead the physics effort can be put into a very targeted review of the changes made.

The Plan Revision Agent, an in‐house‐developed Microsoft Windows (Microsoft Corp., Redmond, WA) service, subscribes to the Plan Revision Events from EventNet. The Plan Revision Agent will extract the current plan's unique identification number (UID) and its predecessor plan UID from the message package passed by EventNet. With the UIDs of the plans, the Plan Revision Agent relies on three .Net libraries, AriaDataProvider, PlanComparisonServiceProvider, and HtmlToPDF, to accomplish the three major tasks separately: 1) get the current plan and predecessor plan in XML format, 2) compare the XML data of the two plans with help of XSLT (EXtensible Stylesheet Language),^(10)^ and 3) represent the comparison result as a PDF report. The output of the Plan Revision Agent is an email with a link to the PDF report that is sent to the clinical physics group. From there, the report can be easily reviewed and added to the patient's record to show proper pretreatment QA for the revised plan.

The Plan Revision Agent has improved the safety and efficiency of reviewing revised plans. In addition, the use of the tool also allows for revisions of intensity modulated treatments without additional measurement‐based QA when compared to a plan that has previous passed QA. This results in considerable time savings. In the previous six months, the monthly range was 21 to 46 plan revisions per month (~ up to 10% of the planning workload).

### C. Mobius Control Agent (MCA)

The MOBIUS3D (Mobius Medical Systems, LP, Houston, TX) software has been utilized in the department as a second‐check QA software of our treatment plans. It requires RTPLAN, RTDOSE, RTSTRUCT, and CT images to perform independent dose calculations and several other QA checks. It automatically imports the DICOM data that is manually exported by the dosimetrists from the treatment planning system (TPS) once the plan is ready for final checks. The dosimetrists export the DICOM data once they have set the plan status to “Planning Approved.” An automatic process was developed to replace the manual exporting process.

**Figure 3:**
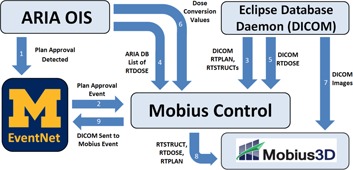
Diagram of the Mobius Control Agent. The nine steps to perform the secondary calculation happen automatically without user interaction.

The MOBIUS Control Agent runs as a Microsoft Windows service that subscribes to the “Plan Approval” plan status change event (Steps 1 and 2) in Fig. 3. The plan and the related RTSTRUCT files are retrieved in DICOM form from the Varian Eclipse DICOM DB daemon (Step 3). The ARIA OIS is queried for the RTDOSE that references this plan, which is also retrieved as DICOM (Step 5). The plan is converted to absolute dose in Gray as required by MOBIUS3D, then the conversion parameters are retrieved from ARIA and the dose files are modified (Step 6). The list of image series is extracted from the RTSTRUCT files, and the Eclipse DB daemon is used to send them directly to Mobius3D (Step 7). The datasets are sent to MOBIUS3D (Step 8), and an event is published via EventNet to indicate that processing is done (Step 9). The automated process improved overall efficiency of the workflow by eliminating the manual DICOM export process. During the time when the process was not automated, data tracking by the physicist checking plans found that plans were not exported for at least one to three plans per day (∼10%–30% of plans completed daily). For a similar four‐month period before and after the MCA was turned on, 31 issues were reported where the plan export was missed or incomplete, whereas after the MCA was activated only three issues were reported. The impact is likely higher as not all failures to export were reported.

### D. Session Audit Agent (SAA)

The Session Audit Agent is a software tool that audits the treatment history data resulting from a patient treatment against the plan data from the OIS. This is similar to the electronic checks developed by others^(11)^ with the addition of an automatic trigger. A typical weekly physics chart check involves a manual review of the recorded treatment history parameters against the planned parameters. This task is well suited for automation by a computer software agent. SAA verifies the parameters and reports any variances to physics. All passing audits are quietly logged in a database and no alert is sent out.

SAA is running on our development system and is in preclinical testing. It reliably audits treatment histories within 30 to 90 s of the end of treatment for the average of 131 patient plans that are treated daily. It is anticipated that, after SAA goes into clinical use, the weekly chart check process will be revised. SAA audits will be able to be followed up immediately regarding any variation in the patient's treatment history instead of waiting for the weekly chart check to catch a possible error. Other parts of the check will continue to be done directly by the physicist, such as a review of the patient's prescription, plan, imaging, and notes between the patient care team. This represents a great benefit to patient care and a time savings for physicists. It replaces a fallible manual audit performed weekly with an algorithm‐based audit that executes soon after each treatment has finished.

### E. Machine Audit Agent (MAA)

Unauthorized or unintended changes of the machine configuration used by the treatment planning system can create unsafe situations. If a needed accessory is removed from the database or machine motion limits are changed, this can adversely impact workflow and patient safety. In an effort to detect these changes, a nightly snapshot of the linear accelerator and high‐dose‐rate brachytherapy (HDR) machine configurations is generated by creating an XML version of selected tables in the OIS. This snapshot is compared to the previous day's snapshot and any differences are reported via an email message to physicists.

To date, the machine audit agent has only triggered reports for preapproved changes to the machine configuration. It correctly reports changes in machine operating limits, along with changes to the trays and accessories. Changes to the HDR machine configuration triggered by a source exchange are reported, as well. This provides a useful backup and review of preapproved changes. MCA is triggered daily at 7 p.m. It is possible that the machine configuration could be changed after the daily audit and changes wouldn't be identified until the next daily check.

### F. Migration Check Tool (MCT)

It is imperative to check plan data when the OIS is upgraded and migrated to a new version of the software and new database. We have previously reported on our Migration Check Tool (MCT) to verify data for treatment plans in the planning or delivery process after the data has been moved to the new database and OIS install.^(12)^ The tool requires that the previous install of the OIS be running on a separate server after the upgraded OIS has finished migration and is released for testing. The tool then compares all of the active plans in the OIS using the original version as the reference and the upgraded version as the test. It uses the same plan comparison method as the Plan Revision Agent.

Our MCT was able to verify 358 plans in 89 min after a major upgrade to our planning system and OIS from Aria 8.9 to Aria 11. Included in the migration were “one of everything” plans that include every combination of X‐ray and electron energy with every combination of treatment accessory. No deviations in plan parameters were found in any of the 358 plans. Selected QA and patient plans were chosen for live “mode up” tests and repeat IMRT QA. Prior to the creation of this software tool, all plans were verified manually at the treatment machines and IMRT QA was repeated on all dynamic treatments. The OIS could not be clinically released until these checks passed. The use of this tool has resulted in more thorough checks and decreased clinical downtime when performing upgrades.

### G. Plan Check Tool (PCT)

The goal of the Plan Check Tool is to support quality treatment plans for physician review and to improve the consistency and efficiency of plan and monitor unit checks prior to the start of a patient's treatment. A framework was developed to support the creation of automated check modules, called checkers, for an overall quality check. Automatic checkers were developed using the application‐programming interface (API) in the TPS (Varian Eclipse) and with custom SQL queries to extend beyond the API's reach. Key design features include the ability to add checkers; configure the rules for a pass, fail, or flag of a check; document additional notes; and generate a report that can be approved and stored into the patient's record. The software was designed to include both manual and automatic checks such that separate treatment‐type specific checklists are no longer required in addition to the Plan Check Tool. The primary users of the tool are dosimetrists to validate the plan before physician review and physicists after physician review to then perform the final approval for treatment. By performing the checks early in the planning process, deviations from planning guidelines and policies can be fixed early in the process, potentially eliminating rework and re‐approvals.

Automatic checkers were created for verifying naming conventions and treatment parameters. For example, dataset, plan, and course names are reported and flagged if they do not follow in‐house‐developed rules. Calculation model information, the presence of bolus, and the presence of an accessory are reported for inspection by the physicist. Prescription information, which is independently entered but redundant between the planning software and OIS, is reported and flagged if in disagreement. Users inspect the reported results of the automatic checks and perform the remaining manual checks. Any flagged items are noted for inclusion in the final report. The graphical user interface allows the user to choose to automatically upload the report using the Document Service in the OIS to the patient's record. The user is then able to complete the document approval process and the report can be viewed by any member of the patient care team.

### H. Winston‐Lutz Agent (WLA)

A Winston‐Lutz test is a standard QA step to check radiation and machine isocenter coincidence before intracranial radiosurgery.^13^, ^14^, ^15^ This test can be time‐consuming; therefore methods were sought to reduce the time required to perform the Winston‐Lutz test through automation. This thereby reduces patient wait times and improves clinical efficiency.

The radiation fields of the Winston‐Lutz test (four 2 cm×2 cm beams at the cardinal gantry angles) were created in a test patient for repeated use. For each field, MV portal images are acquired of a target ball, which is placed at the isocenter using the in‐room lasers. The WLA is subscribed to the “Session Complete” event for this specific QA plan in the OIS. Images are automatically retrieved and the field edges and target ball are found through image processing. The displacement between the radiation isocenter and the target ball is calculated and a report is generated.

**Figure 4:**
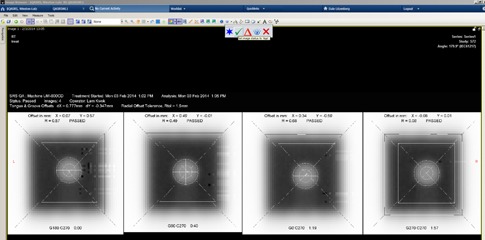
Results of Winston‐Lutz Agent pushed into the machine QA record in Aria for review and approval using the OIS approval method.

Graphical and numerical results are posted to a website viewable at the treatment machine. Results are also rendered as a DICOM image sent back to the ARIA OIS to the machine QA patient record for documentation and approval, as shown in Fig. 4. The updated process requires 4 min, whereas the previous manual method required 25 min.

The results were found to be more accurate and consistent than the previous method. The images were also analyzed with DoseLab (Mobius Medical Systems) for comparison. The difference between the film and automated Winston‐Lutz results in the X and Y direction and the radius were (−0.17±0.28) mm, (0.21±0.20) mm, and (−0.14±0.27) mm, respectively. The difference between the DoseLab and automated Winston‐Lutz results were (−0.05±0.06) mm, (−0.01±0.02) mm, and (0.01±0.07) mm, respectively. Accuracy and consistency of results were improved over the previous method and were comparable to other commercial solutions.

## III. RESULTS AND DISCUSSION

The use of small, focused teams consisting of one or more clinical physicists and software engineers was essential to the success of these QA‐ and patient safety‐related projects. This reduced the communication loop needed to complete projects and fostered working collaborations to speed the development of future projects. Over time, software engineers gained experience in specific software tools reducing development time. This also facilitated the needed testing, commissioning, training, roll out, and support of these tools.

We have significantly reduced the time necessary to perform critical QA steps in radiotherapy. The easiest item to automate was transfer of data and parameter checking. To supplement the vendor supplied tools we had to develop our own Web service applications to obtain plan information from the OIS. This was used in the PRA, SAA, and MCT.

There have been numerous efforts over the years to create more quantitative, robust, and electronic checks of the RT process. Siochi et al.^(16)^ reported on an advanced reporting tool for plan checks in a paperless system. Santanam et al.^(17)^ reported on the importance of enforcing standards in the naming conventions in RT plans. We combined this with our lean thinking process to develop the Plan Check Tool to report on critical aspects of the plan and enforce naming conventions in our clinic to create a uniform output for all plans.

Many of our tools focus on tasks such as checking and validating plan data at critical time points.^(18)^ This is similar to the IHE‐RO Quality Assurance with Plan Veto (QAPV) profile.^(19)^ Noel et al.^(20)^ used an incidence learning system to show that implementation of the QAPV profile could significantly reduce the RPN values of an FMEA analysis of their RT process. We implemented aspects of a QAPV workflow for new plans with MCA, PRA, MCT, and SAA.

The event‐driven framework greatly facilitated the automatic workflow of the QA tasks. Data sources outside our control (e.g., Aria OIS) required an EventFinder to watch the database for changes to generate and publish events. Adding more data sources would require the source to be open to queries so that our in‐house‐developed EventFinder would work. Data sources that are closed to the user and do not allow users to access their data cannot benefit from this technique. The EventNet system also requires extra servers beyond the OIS. This would be difficult for the average clinic to implement and may require a vendor‐supplied solution for wide adoption.

## V. CONCLUSIONS

An event‐driven framework is a major breakthrough for automating the RT process. We are at the beginning of an effort to invert the data chase in radiotherapy so that critical QA processes are triggered by milestones in the RT planning and delivery process with only the important results being passed to the users. We expect that a clinic looking to implement adaptive radiotherapy will require this type of event‐driven framework to perform the necessary QA within a limited amount of time. This should allow us to keep our focus on patient care, high quality plans, and safe radiation therapy delivery.

## ACKNOWLEDGMENTS

This work partially supported under NIH Contract P01CA059827.

## References

[1] Moran JM , Dempsey M , Eisbruch A , et al. Safety considerations for IMRT: Executive summary [Internet]. Pract Radiat Oncol. 2011;1(3):190–95. Available from: http://linkinghub.elsevier.com/retrieve/pii/S1879850011001627?showall=true 25740119 10.1016/j.prro.2011.04.008 PMC3808751

[2] Bogdanich W . Radiation offers new cures, and ways to do harm. The New York Times [Internet]. New York; 2010 Jan 23; Sect. Health. Available from: http://www.nytimes.com/2010/01/24/health/24radiation.html?pagewanted=all

[3] Perry H , Mantel J , Lefkofsky MM . A programmable calculator to acquire, verify and record radiation treatment parameters from a linear acceleration. Int J Radiat Oncol Biol Phys. 1976;1(9‐10):1023–26. 824255 10.1016/0360-3016(76)90133-4

[4] Podmaniczky KC , Mohan R , Kutcher GJ , Kestler C , Vikram B . Clinical experience with a computerized record and verify system. Int J Radiat Oncol Biol Phys. 1985;11(8):1529–37. 4019277 10.1016/0360-3016(85)90342-6

[5] Fraass BA , Lash KL , Matrone GM , et al. The impact of treatment complexity and computer‐control delivery technology on treatment delivery errors. Int J Radiat Oncol Biol Phys. 1998;42(3):651–59. 9806527 10.1016/s0360-3016(98)00244-2

[6] Huang G , Medlam G , Lee J , et al. Error in the delivery of radiation therapy: results of a quality assurance review. Int J Radiat Oncol Biol Phys. 2005;61(5):1590–95. 15817367 10.1016/j.ijrobp.2004.10.017

[7] Leunens G , Verstraete J , Van den Bogaert W , Van Dam J , Dutreix A , van der Schueren E . Human errors in data transfer during the preparation and delivery of radiation treatment affecting the final result: “garbage in, garbage out.” Radiother Oncol. 1992;23(4):217–22. 1609125 10.1016/s0167-8140(92)80124-2

[8] Kim CS , Hayman JA , Billi JE , Lash K , Lawrence TS . The application of lean thinking to the care of patients with bone and brain metastasis with radiation therapy. J Oncol Pract. 2007;3(4):189–93. 20859409 10.1200/JOP.0742002 PMC2793825

[9] Pawlicki T , Dunscombe P , Mundt AJ , Scalliet P . Quality and safety in radiotherapy. Boca Raton: CRC Press; 2010.

[10] XSLT Working Group. The Extensible Stylesheet Language Family (XSL) [Internet]. Accessed 2015 Sep 15. Available from: http://www.w3.org/Style/XSL/

[11] Li HH , Wu Y , Yang D , Mutic S . Software tool for physics chart checks. Pract Radiat Oncol. 2014;4(6):e217–25. 25407872 10.1016/j.prro.2014.03.001

[12] Hadley SW , White D , Chen X , Moran JM , Keranen WM . Migration check tool: automatic plan verification following treatment management systems upgrade and database migration. J Appl Clini Med Phys. 2013;14(6):4394. 10.1120/jacmp.v14i6.4394 PMC5714629 24257281

[13] Lutz W , Winston KR , Maleki N . A system for stereotactic radiosurgery with a linear accelerator. Int J Radiat Oncol Biol Phys. 1988;14(2):373–81. 3276655 10.1016/0360-3016(88)90446-4

[14] Rowshanfarzad P , Sabet M , O'Connor DJ , Greer PB . Verification of the linac isocenter for stereotactic radiosurgery using cine‐EPID imaging and arc delivery. Med Phys. 2011;38(7):3963–70. 21858993 10.1118/1.3597836

[15] Winey B , Sharp G , Bussière M . A fast double template convolution isocenter evaluation algorithm with subpixel accuracy. Med Phys. 2011;38(1):223–27. 21361190 10.1118/1.3524227

[16] Siochi RA , Pennington EC , Waldron TJ , Bayouth JE . Radiation therapy plan checks in a paperless clinic. J Appl Clin Med Phys. 2009;10(1):2905. 19223840 10.1120/jacmp.v10i1.2905 PMC5720497

[17] Santanam L , Hurkmans C , Mutic S , et al. Standardizing naming conventions in radiation oncology. Int J Radiat Oncol Biol Phys. 2012;83(4):1344–49. 22245204 10.1016/j.ijrobp.2011.09.054 PMC4306340

[18] Siochi RA , Balter P , Bloch CD , et al. A rapid communication from the AAPM Task Group 201: recommendations for the QA of external beam radiotherapy data transfer. AAPM TG 201: quality assurance of external beam radiotherapy data transfer. J Appl Clin Med Phys. 2010;12(1):3479. 21330992 10.1120/jacmp.v12i1.3479 PMC5718574

[19] Integrating Healthcare Enterprise (IHE). Quality Assurance with Plan Veto [Internet]. 2014 Accessed 2015 Feb 26. Available from: http://www.ihe‐ro.org/lib/exe/fetch.php?media=doc:profiles:qapv:ihe‐roqapvsupplement119.doc

[20] Noel CE , VeeraRajesh G , Bosch WD , et al. Quality Assurance with Plan Veto: reincarnation of a record and verify system and its potential value. Int J Radiat Oncol Biol Phys. 2014;88(5):1161–66. Available from: http://www.sciencedirect.com/science/article/pii/S036030161303722X 24661669 10.1016/j.ijrobp.2013.12.044

